# Prognostic significance of anti-p53 and anti-KRas circulating antibodies in esophageal cancer patients treated with chemoradiotherapy

**DOI:** 10.1186/1471-2407-12-119

**Published:** 2012-03-26

**Authors:** Pierre Blanchard, Laurent Quero, Vincent Pacault, Marie-Helene Schlageter, Valerie Baruch-Hennequin, Christophe Hennequin

**Affiliations:** 1 Radiation Oncology Department, Hôpital Saint Louis, AP-HP, Paris, France; 2 Radiation Oncology Department, Institut Gustave Roussy, Villejuif, France; 3 Unité de Biologie Cellulaire, Hôpital Saint Louis, AP-HP, Paris, France; 4 Radiation Oncology Department, Hôpital Saint Louis, 1, Avenue Claude Vellefaux, Paris 75010, France

**Keywords:** Esophageal cancer, Radiotherapy, Chemotherapy, p53, Ras

## Abstract

**Background:**

P53 mutations are an adverse prognostic factor in esophageal cancer. P53 and KRas mutations are involved in chemo-radioresistance. Circulating anti-p53 or anti-KRas antibodies are associated with gene mutations. We studied whether anti-p53 or anti-KRas auto-antibodies were prognostic factors for response to chemoradiotherapy (CRT) or survival in esophageal carcinoma.

**Methods:**

Serum p53 and KRas antibodies (abs) were measured using an ELISA method in 97 consecutive patients treated at Saint Louis University Hospital between 1999 and 2002 with CRT for esophageal carcinoma (squamous cell carcinoma (SCCE) 57 patients, adenocarcinoma (ACE) 27 patients). Patient and tumor characteristics, response to treatment and the follow-up status of 84 patients were retrospectively collected. The association between antibodies and patient characteristics was studied. Univariate and multivariate survival analyses were conducted.

**Results:**

Twenty-four patients (28%) had anti-p53 abs. Abs were found predominantly in SCCE (p = 0.003). Anti-p53 abs were associated with a shorter overall survival in the univariate analysis (HR 1.8 [1.03-2.9], p = 0.04). In the multivariate analysis, independent prognostic factors for overall and progression-free survival were an objective response to CRT, the CRT strategy (alone or combined with surgery [preoperative]) and anti-p53 abs. None of the long-term survivors had p53 abs. KRas abs were found in 19 patients (23%, no difference according to the histological type). There was no significant association between anti-KRas abs and survival neither in the univariate nor in the multivariate analysis. Neither anti-p53 nor anti-KRas abs were associated with response to CRT.

**Conclusions:**

Anti-p53 abs are an independent prognostic factor for esophageal cancer patients treated with CRT. Individualized therapeutic approaches should be evaluated in this population.

## Background

Esophageal cancer is a public health issue worldwide. Its incidence has remained stable over the past thirty years but its pathologic features have changed dramatically. The incidence of adenocarcinoma of the esophagus (ACE) increased 4-fold during this period in the United States, while that of squamous cell carcinoma (SCCE) declined by 30%. In contrast, the incidence of SCCE is highest in Asia, southern and eastern Africa, and northern France, with an annual mortality rate approximating 100 per 100,000 [1]. The risk factors, coexisting conditions, location in the esophagus, natural history and survival differ between these two histological subtypes. Yet despite these differences, therapeutic strategies are very similar and combine surgery, chemotherapy and radiotherapy [2]. In locally advanced disease, concomitant chemoradiation (CRT) is the standard treatment [3,4].

Major prognostic factors for esophageal carcinomas include clinical factors (general condition, initial weight loss, baseline hemoglobin level), factors related to local spread (TNM stage [5], lymph node micrometastases, the ratio between involved and sampled nodes, extracapsular lymph node involvement), and factors related to the radicality of surgery or to response to medical treatment evaluated radiographically or endoscopically. These factors were recently reviewed for ACE [6]. In addition, molecular pathology has revealed numerous genes and molecules associated with tumor invasion and metastasis, some of which exert a prognostic impact *per se*. A better knowledge of these factors may not only improve prognostication but may offer new individually tailored therapeutic options [7].

Esophageal carcinogenesis is a multi-step process that transforms normal human cells into tumor cells following multiple genetic alterations. The circumvention of apoptosis appears to play an early and central role in this process. Mutations of the p53 gene are responsible for reduced chemo- and radio-induced apoptosis in esophageal cancer [8]. These mutations are present in around 50% of esophageal cancers, and associated with advanced-stage disease, poor response to CRT and shorter survival [9,10]. It has even been suggested that the analysis of p53 polymorphisms performed on endoscopic biopsies could identify patients with Barrett's esophagus who are at risk of neoplastic progression. It could therefore complement the histological examination in deciding the frequency of endoscopic surveillance in this population.

KRas is a membrane-bound guanosine triphosphate (GTP)/guanosine diphosphate (GDP)-binding (G) protein that serves as a "molecular switch," converting signals from the cell membrane to the nucleus. These chemical signals lead to protein synthesis and the regulation of cell survival, proliferation, and differentiation. It is mutated in 30% of solid tumors, and particularly in 95% of pancreatic cancers and 50% of colon cancer, but probably only in less than 10% of esophageal tumors [11]. However, some reports have suggested much higher levels of mutations in esophageal carcinomas. In colon cancer, Ras mutations have been shown to be predictive of resistance to anti-EGFR therapy [12].

Gene mutations lead to the synthesis and accumulation of physically altered proteins that are recognized as « non-self ». The immune system develops antibodies directed against these aberrant proteins. The detection of serum antibodies specific for p53 or KRas could be an easy way to determine an individual's mutational status. The aim of this study was to investigate whether the level of serum anti-p53 and anti-KRas antibodies measured prior to CRT is a prognostic marker in esophageal carcinoma patients treated with CRT.

## Methods

### Patients

We retrospectively reviewed the files of consecutive patients treated with CRT for esophageal cancer (SCCE or ACE) at Saint Louis University Hospital (Paris, France) between December 1999 and December 2002. Data were collected in 2007 and included: age, sex, performance status, tumor stage according to TNM and International Union against Cancer (UICC) classifications, presence of dysphagia, weight loss, baseline hemoglobin level, type of treatment, treatment-related toxicities. Patients had been followed up every three months after completion of treatment and regularly assessed for the evaluation of response, local or distant relapse and their vital status. The vital status had last updated in July 2009. The use of the patients' clinical data was approved by the local research and ethics committee.

### Treatment

Treatment consisted of concomitant CRT. Radiation doses ranging from 40 Gy (peri-operative radiation therapy) to 66 Gy (definitive radiotherapy) were adapted to each patient's treatment. Therapy had been delivered using 3D conformal planning and conventional fractionation (1.8 Gy per day, five days a week). Unless contraindicated, concomitant chemotherapy had been administered and consisted of cisplatinum (100 mg.m^-2^, day 2) and 5-Flurouracil (1000 mg.m^-2^.j^-1^, continuous infusion for 3 days), repeated every 4 weeks. Treatment had been followed by two to three cycles of Cisplatinum-5FU-based chemotherapy. When tumors were considered potentially resectable, tumor response had been evaluated after 40 Gy and operable patients had undergone surgery in case of a good response. Some patients who had initially undergone curatively-intended surgery had received CRT due to the presence of pathologic features carrying a poor prognosis. Response and follow-up included repeated CT scans and esophagoscopies. Complete response was defined as a normal CT scan and esophagoscopy with negative biopsies.

### Enzyme Immunoassay for p53 Abs and KRas Abs

Serum anti-p53 and anti-KRas Abs had been measured in each patient during the week before the initiation of CRT. For the 6 patients who had received post-operative radiotherapy, antibodies had been measured after surgery. Anti-p53 Abs were assessed by ELISA with the anti-p53 ELISA Kit II (Pharmacell^®^, Paris, France) and anti-KRas Abs by a non-commercialized Pharmacell^® ^kit. The method used is an Enzyme Linked Immuno-Sorbent Assay (ELISA) using microtiter plates coated with recombinant wild-type human p53 or KRas protein (to detect specific anti-p53 or anti-KRas antibodies) or with control proteins (to detect non-specific interactions). A peroxidase-conjugated goat anti-human IgG binds to auto-antibodies. The specific protein/auto-antibody/conjugate complexes are revealed by adding a peroxidase substrate which results in a colorimetric reaction. Quality procedures included three control tests for each measurement which was scored as: strong positive, mild positive and negative. Auto-antibody values are expressed in arbitrary units (AU). For anti-p53 auto-antibodies, the cut-off value for positivity was set at the average value among healthy subjects plus three standard deviations (anti-p53) or plus one standard deviation (anti-KRas), that is 1.15 U/mL for anti-p53 abs and 0.25 U/smL anti-KRas abs. These values had previously been determined by Pharmacell^®^. Physicians had been blinded to the results of anti-p53 and anti-KRas antibody tests during the treatment period and follow-up, which had been disclosed by one investigator (MHS) after the end of all treatments.

### Statistical analysis

Percentages were compared using the chi-square or Fisher's exact test when appropriate. Quantitative variables were compared using the Student *t *test or the Mann-Whitney rank test. Follow-up was estimated using the reverse Kaplan-Meier method [13]. Correlation between p53 and KRas auto-antibody values was estimated using Pearson's correlation coefficient. Overall survival and disease-free survival were estimated using the Kaplan-Meier method. Progression-free survival was defined as the time between the beginning of radiotherapy to relapse or death, whichever occurred first. Survival curves were compared using the logrank test for the univariate analysis. Variables associated with disease-free or overall survival with a p-value < 0.25 were included in a multivariate ascending stepwise Cox regression analysis. In the Cox model, continuous variables were dichotomized. Missing values were rare (< 1.5%) and were therefore omitted. Statistical analyses were performed using SAS software, version 9.1 (SAS Inc, United States). All reported p-values are two-sided, and p-values lower than 0.05 were considered significant.

## Results

### Patients and tumors

Between December 1999 and December 2002, 97 patients were referred for concomitant CRT for esophageal carcinoma. Thirteen of them had major missing values and were therefore excluded from the present analysis. The study population therefore consists of 84 patients. Median follow-up was 87 months (7.2 years, range: 1-107 months). Only three patients (4%) had been lost to follow-up during the first 5 years. Table 1 shows patient and tumor characteristics. The majority of patients were male (75 patients 84%), median age was 60 years (range 38-81 years), in good general condition (Karnofsky performance status 80-100: 67 patients 80%). Weight loss was 10% or more in 49% of the patients. Tumor characteristics were as follows: squamous cell carcinomas (n = 57) and adenocarcinomas (n = 27). Most of the tumors were considered unresectable. Six patients had received post-operative irradiation due to pathological evidence of involved mediastinal lymph nodes on the surgical specimen. Sixteen patients had received pre-operative CRT. For the 17 patients with metastases, CRT had been administered because disease was pauci-metastatic, they were in good general condition and had presented with dysphagia.

**Table 1:** patient and tumor characteristics

		Missing values	n (%)
**Overall population**			84 (100)

**Gender**	Female	0	9 (11)

	Male		75 (84)

**Age (years)**	< 60	0	42 (50)

	≥ 60		42 (50)

**Histology**	ACE	0	27 (32)

	SCCE		57 (68)

**Karnofsky Performance status**	≤ 60	1	10 (12)

	70		6 (7)

	80		21 (25)

	90		25 (30)

	100		21 (25)

**Dysphagia**	Solid	0	64 (76)

	Liquid		20 (24)

**Weight loss ≥ 10%**	No	2	42 (51)

	Yes		40 (49)

**Hemoglobin level < 120 g/L**	Yes	0	57 (68)

	No		27 (32)

**T Stage**	1	0	3 (4)

	2		10 (12)

	3		59 (70)

	4		12 (14)

**N Stage**	0	0	28 (33)

	1		27 (32)

	2		29 (35)

**M Stage**	No	0	67 (80)

	Yes		17 (20)

**Surgery**	No	0	78 (81)

	Yes		16 (19)

**Chemoradiotherapy**	Preoperative	0	16 (19)

	Postoperative		6 (7)

	Alone		62 (74)

*Abbreviations*: *SCCE*, squamous cell carcinoma of the esophagus; *ACE*, adenocarcinoma of the esophagus

### Anti-p53 and anti-KRas antibody levels

The median anti-p53 antibody value was 0.25 (range: 0-159). Anti-p53 Abs were considered positive in 24 patients (29%). All except one patient with positive anti-p53 Abs had squamous cell carcinoma (23/24). The median anti-KRas antibody value was 0.16 (range: 0-1.19). Anti-KRas Abs were considered positive in 19 patients (23%). There was no correlation between the two serum auto-antibody levels (Pearson correlation coefficient: 0.05, p-value = 0.6). The 13 excluded patients had the same levels and frequency of positivity as the study patients for both auto-antibodies (levels: Mann-Whitney rank test, p = 0.7 for anti-p53 and p = 0.5 for anti-KRas; positivity: Chi-square test, p = 0.6 for anti-p53 and p = 0.13 for anti-KRas).

### Association between anti-p53 and anti-KRas antibodies and clinico-pathologic characteristics

The association between antibody positivity and patient characteristics is presented in Table 2. A younger age and SCC histology were associated with positive anti-p53 Abs, whereas no characteristics were associated with anti-KRas positivity. Neither anti-p53 nor anti-KRas antibodies were correlated with an objective response to CRT. Response rates were 67% in anti-p53 negative patients versus 78% in anti-p53 positive patients (p = 0.4), and 65% in anti-KRas negative patients versus 82% in anti-KRas positive patients (p = 0.2).

**Table 2:** Association between anti-p53 and anti-KRas antibodies and clinico-pathologic characteristics (p-values were calculated using the chi-squared test)

Characteristics		Anti-p53 antibody			Anti-KRas antibody		
		**Negative**	**Positive**	**p-value**	**Negative**	**Positive**	**p-value**

**Gender**	Female	6 (10)	3 (12.5)	0.7	9 (14)	0 (0)	0.09

	Male	54 (90)	21 (87.5)		56 (86)	19 (100)	

**Age (years)**	< 60	25 (42)	17 (71)	0.02	32 (49)	10 (53)	0.8

	≥ 60	35 (58)	7 (29)		33(51)	9 (47)	

**Histology**	ACE	25 (42)	2 (8)	0.003	24 (37)	3 (16)	0.08

	SCCE	35 (58)	22 (91)		41 (63)	16 (84)	

**Karnofsky Performance status**	≤ 80	23 (38)	14 (61)	0.06	27 (42)	10 (53)	0.4

	90-100	37 (62)	9 (39)		37 (58)	9 (47)	

**Weight loss ≥ 10%%**	No	33 (57)	9 (37.5)	0.1	34 (53)	8 (44)	0.5

	Yes	25 (43)	15 (62.5)		30 (47)	10 (56)	

**Dysphagia**	Solid	49 (82)	15 (62.5)	0.06	48 (74)	16 (84)	0.4

	Liquid	11 (18)	9 (32.5)		17 (26)	3 (16)	

**Hemoglobin level < 120 g/L**	Yes	40 (37)	17 (29)	0.5	20 (31)	9 (47)	0.2

	No	20 (63)	7 (71)		45 (69)	10 (53)	

**T Stage**	1-2	12 (20)	1 (4)	0.07	12 (18)	1 (5)	0.2

	3-4	48 (80)	23 (96)		53 (82)	18 (95)	

**N Stage**	0	19 (32)	9 (37.5)	0.6	22 (34)	6 (32)	0.9

	1-2	41 (68)	15 (62.5)		42 (66)	16 (68)	

**M Stage**	Non	49 (82)	18 (75)	0.5	52 (80)	15 (79)	0.9

	Oui	11 (18)	6 (25)		13 (20)	4 (21)	

*Abbreviations*: *SCCE*, squamous cell carcinoma of the esophagus; *ACE*, adenocarcinoma of the esophagus

### Survival analysis

**Figure 1:**
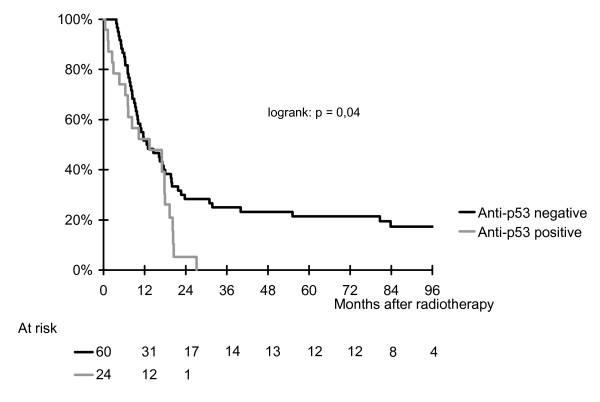
**Kaplan-Meier curve of overall survival according to anti-p53 antibody status**.

**Figure 2:**
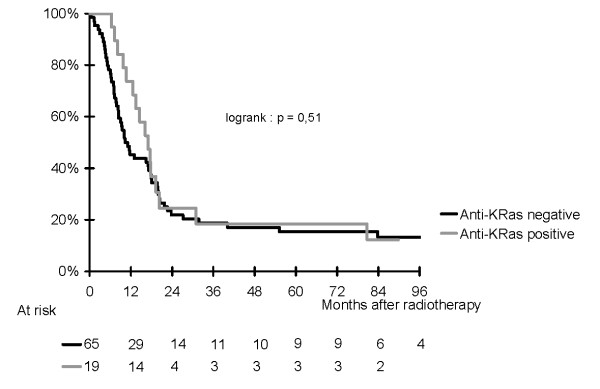
**Kaplan-Meier curve of overall survival according to anti-KRas antibody status**.

Overall 71 patients have died, among which 22 had anti-p53 antibodies and 16 had anti-KRas antibodies. Median overall survival for the entire population was 13 months (95% CI: 719 months. There was a difference according to the anti-p53 status, in favor of patients with a negative anti-p53 abs (HR for death [95% CI]: 1.8 [1.03, 2.9], logrank p = 0.04). None of the 12 long-term survivors had p53 antibodies. KRas antibodies were not associated with overall survival (HR for death [95% CI]: 0.8 [0.5, 1.5], logrank p = 0.5). Figures 1 and 2 show Kaplan-Meier curves for overall survival for anti-p53 and anti-KRas antibodies.

### Univariate and multivariate analyses

Table 3 summarizes the results of the univariate and multivariate analyses. Factors associated with improved overall survival in the univariate analysis were age > 60 years, a good performance status, no visceral metastases, an objective response to CRT, preoperative CRT followed by surgery and no anti-p53 antibodies. After the multivariate analysis, the factors independently associated with improved overall survival were: preoperative CRT, an objective response to CRT and no anti-p53 antibodies. The progression-free survival analysis showed similar results, as most relapses were shortly followed by death. Factors independently associated with better progression-free survival were: preoperative CRT, an objective response to CRT and no anti-p53 antibodies, as shown in Table 4.

**Table 3:** Univariate analysis of prognostic factors for overall survival

	Univariate analysis (Logrank)			Multivariate analysis (Cox)		
**Characteristics (reference)**	**HR**	**95% CI**	***P-value***	**HR**	**95% CI**	***P-value***

**Patient Characteristics**						
**Gender (male vs female)**	1.3	0.6-2.7	0.6	-	-	-
**Age (≥ 60 vs < 60 years)**	0.5	0.3-0.8	0.005	-	-	-
**Pretreatment hemoglobin level (< 120 vs ≥ 120 g/L)**	0.97	0.6-1.6	0.9	-	-	-
**Initial weight loss (≥ 10% vs < 10%)**	1.4	0.8-2.2	0.2	1.5	0.9-2.7	0.1
**Dysphagia (mild vs liquid)**	1.3	0.8-2.3	0.3	-	-	-
**Karnofsky Performance status (≤ 80 vs 90-100)**	2	1.25-3.3	0.008	1.7	0.99-3	0.06

**Tumor Characteristics**						
**Histology (SCCE vs ACE)**	1.15	0.7-1.9	0.6	-	-	-
**T Stage (3-4 vs 1-2)**	1.1	0.6-2	0.8	-	-	-
**N Stage (1 vs 0)**	1.4	0.9-2.4	0.15	-	-	-
**M stage (1 vs 0)**	1.8	1.01-3.1	0.04	-	-	-

**Treatment-related Characteristics**						
**Objective response to CRT (no vs yes)**	3.3	1.9-5.9	< 0.0001	3.3	1.6-6.8	< 0.001
**Definitive CRT vs Preoperative CRT**	2.5	1.4-5.0	0.005	7.1	3.6-13.1	< 0.0001

**Antibodies**						

**Anti-p53 (positive vs negative)**	1.8	1.03-2.9	0.04	2	1.05-2.8	0.04
**Anti-Ras (positive vs negative)**	0.8	0.5-1.5	0.5	-	-	-

*Abbreviations*: *ACE*, adenocarcinoma of the esophagus; *CI*, confidence interval; *CRT*, chemoradiotherapy; *HR*, hazard ratio; *SCCE*, squamous cell carcinoma of the esophagus

**Table 4:** Univariate analysis of prognostic factors for progression-free survival

	Univariate analysis(Logrank)			Multivariate analysis (Cox)		
**Characteristics (reference)**	**HR**	**95% CI**	***P- value***	**HR**	**95% CI**	***P-value***

**Patient Characteristics**						
**Gender (male vs female)**	1.4	0.6-3.5	0.4	-	-	-
**Age (> 60 vs < 60 years)**	0.5	0.3-0.8	0.02	0.6	0.3-1.1	0.1
**Pretreatment hemoglobin level (< 120 vs > 120 g/L)**	1	0.6-1.6	0.9	-	-	-
**Initial weight loss (> 10% vs < 10%)**	1.3	0.8-2.1	0.3	-	-	-
**Dysphagia (mild vs liquid)**	1.2	0.7-2.1	0.4	-	-	-
**Karnofsky Performance status (< 80 vs 90-100)**	0.6	0.3-0.9	0.01	-	-	-

**Tumor Characteristics**						
**Histology (SCCE vs ACE)**	1	0.6-1.7	0.9	-	-	-
**T Stage (3-4 vs 1-2)**	1	0.5-1.8	0.9	-	-	-
**N Stage (1 vs 0)**	1.3	0.8-2	0.4	-	-	-
**M stage (1 vs 0)**	1.8	1-3.1	< 0.05	1.7	0.9-3.4	0.1

**Treatment-related Characteristics**						
**Objective response to CRT (no vs yes)**	3.3	2.0-5.0	< 0.0001	4.8	2.4-9.4	< 0.0001
**Definitive CRT vs Preoperative CRT**	2.5	1.25-5.0	0.01	2.4	1.2-4.8	< 0.02

**Antibodies**						

**Anti-p53 (positive vs negative)**	1.8	1.05-2.9	0.03	2	1-3.9	0.04
**Anti-Ras (positive vs negative)**	0.8	0.5-1.4	0.5	-	-	-

*Abbreviations*: *ACE*, adenocarcinoma of the esophagus; *CI*, confidence interval; *CRT*, chemoradiotherapy; *HR*, hazard ratio; *SCCE*, squamous cell carcinoma of the esophagus

## Discussion

This retrospective study is the first to evaluate the prognostic significance of anti-p53 and anti-KRas antibodies in esophageal carcinoma patients treated with CRT. Anti-p53 antibodies were found in 24 patients (29%), mainly in SCCE (23/24 patients). Anti-KRas antibodies were found in 19 patients (23%). It shows that the presence of serum anti-p53 antibodies measured prior to CRT is an independent prognostic factor in esophageal carcinoma treated with CRT. None of the long-term survivors had anti-p53 antibodies. It is one of the largest studies to evaluate the prognostic value of anti-p53 antibodies in esophageal cancer. In our series anti-KRas antibodies had no prognostic impact on esophageal cancers. Response to CRT and preoperative CRT were the other independent prognostic factors, which are even more strongly correlated with survival. Anti-p53 antibodies were mostly restricted to SCCE, suggesting that they could have a lower prognostic impact on ACE. There was no correlation between anti-p53 antibodies and response to CRT. Thus the prognostic impact of anti-p53 abs is independent of the initial response to CRT, but most probably stems from a lower rate of relapse in anti-p53-negative tumors.

According to our study, anti-KRas antibodies have no prognostic value in esophageal carcinoma, which could be linked to the low percentage of *ras *mutations in this cancer. However, their prognostic value should be evaluated in other cancers where *ras *mutations are more frequent.

The major limitations of this study are related to its retrospective and monocentric nature. This series appears heterogeneous because various locoregional treatments were administered. In addition, there may be a selection bias due to the exclusion of 13 patients because of missing patient information. However, such a bias is less likely because auto-antibody values were similar between the excluded and included populations. Moreover, all known clinical prognostic factors [6] were collected for the study population, thus minimizing confusion bias. As recommended in biomarker studies, the treating physicians were blinded to the auto-antibody results, which therefore had no incidence on the therapeutic strategy. As with any retrospective study, this series needs to be confirmed by other retrospective and if possible prospective studies. Table 5 shows the prognostic value of p53 mutations for overall survival [8-10,14-16], event-free survival [9,17] and response rate [8,9,18] in other reports on esophageal cancer. Those values are consistent with the findings reported here. They are also consistent with series reported concerning other cancers [for review see [19].

**Table 5:** p53 status and prognosis of esophageal carcinoma: comparison between literature data and the present report

Reference	n	Histology	Treatment	Methods for determining p53 status				Frequency of altered p53 pathway	Prognostic value		
				**Antibody**	**IHC**	**Sequencing**	**Function**		**Overall survival**	**Event-Free survival**	**Response**

**Ireland [**10**]**	37	ACE	Surg	**-**	**-**	**+**	**-**	49% (S)	Univariate	NA	NA
**Hagiwara **[20]	42	SCCE	Surg	**+**	**+**	**-**	**-**	28% (Ab) 70% (IHC)	NS	NA	NA
**Ribeiro **[9]	42	ACE/SCCE	CT-CRT-Surg	**-**	**+**	**+**	**-**	59% (IHC) 40% (S)	Univariate (S but not IHC)	Univariate (S but not IHC)	Correlation
**Takahashi **[17]	44	SCCE	Surg	**+**	**-**	**-**	**-**	36% (Ab)	NA	Univariate	NA
**Shimada **[21]	35	SCCE	Surg	**+**	**+**	**-**	**-**	40% (Ab) 54% (IHC)	NA	NA	NA
**Shimada **[16]	110	SCCE	Surg	**+**	**-**	**-**	**-**	36% (Ab)	Multivariate (if p53-Ab disappear post Surg)	NA	NA
**Berqvist **[14]	34	SCCE/ACE	CRT - CT	**+**	**-**	**-**	**-**	21% (Ab)	Multivariate	NA	NA
**Cai **[18]	46	SCCE	CRT	**+**	**-**	**-**	**-**	39% (Ab)	NA	NA	Correlation
**Kihara **[8]	138	SCCE	Surg	**-**	**-**	**+**	**-**	56.5% (S)	Multivariate	NA	Correlation
**Schneider **[15]	59	ACE	Surg	**-**	**-**	**+**	**-**	51% (S)	Multivariate	NA	NA

**Present Study**	84	SCCE/ACE	CRT +/- Surg	**+**	**-**	**-**	**-**	29% (Ab)	Multivariate	Multivariate	NS

*Abbreviations*: *Ab*, antibody; *ACE*, adenocarcinoma of the esophagus; *CRT*, chemoradiotherapy; *CT*, chemotherapy; *IHC*, immunohistochemistry; *NS*, not significant; *NA*, not available; *S*, sequencing; *SCCE*, squamous cell carcinoma of the esophagus

The methods used to determine the p53 or *k-ras *mutational status merit discussion. Indeed, the question is whether anti-p53 antibodies are a reliable yardstick for the p53 mutational status. Many studies previously demonstrated that p53 antibodies were restricted to cancer patients bearing p53 mutations [22,23]. These antibodies have a high specificity, but lack sensitivity. Indeed, they have the same drawbacks as immunohistochemistry, because they are absent in patients in whom p53 mutations result in the absence of p53 protein synthesis and accumulation [24]. Other methods such as sequencing, immunohistochemical analysis and functional assays have been developed to determine the p53 status and have been applied to esophageal carcinomas (Table 5). Nowadays DNA chips are being used increasingly to investigate the tumor genetic status. As they allow many genes to be studied simultaneously, we may no longer need to use tests like anti-p53 antibodies. Notwithstanding, measuring serum antibodies with the ELISA method is easy and reproducible. This could be a simple way to determine the p53 status, with quantitative information. Indeed, the perioperative variations in serum p53 antibodies have been shown to predict overall survival [16]. P53 antibody titers could be used for the follow-up of patients with initially elevated p53 antibodies. As p53 mutated tumors have a worse prognosis and different response to treatment than p53 wild type tumors, monitoring the p53 tumor status and function is central in the context of individualized medicine.

## Conclusions

In brief, our study shows that anti-p53 antibodies are an independent prognostic marker in esophageal cancer patients. The antibodies are mostly restricted to squamous cell carcinoma. This series is consistent with most published series studying the p53 status in esophageal cancer, suggesting that the p53 status should be monitored in esophageal cancer. Larger-scale studies are needed to better define the prognostic implications of p53 mutations, their distribution amongst the histological subtypes of esophageal cancer, and whether treatments should be adapted to the tumor p53 status.

## Competing interests

The authors declare that they have no competing interests.

## Authors' contributions

PB, CH and MHS participated in the design of the study, PB performed the statistical analysis. All authors participated in the coordination. PB and CH drafted the manuscript. All authors read and approved the final manuscript.

## Pre-publication history

The pre-publication history for this paper can be accessed here:

http://www.biomedcentral.com/1471-2407/12/119/prepub

## References

[B1] Holmes RS Vaughan TL Epidemiology and pathogenesis of esophageal cancer Semin Radiat Oncol 2007 17 2 9 10.1016/j.semradonc.2006.09.003 17185192

[B2] Mariette C Piessen G Triboulet JP Therapeutic strategies in oesophageal carcinoma: role of surgery and other modalities Lancet Oncol 2007 8 545 553 10.1016/S1470-2045(07)70172-9 17540306

[B3] Bedenne L Michel P Bouche O Chemoradiation followed by surgery compared with chemoradiation alone in squamous cancer of the esophagus: FFCD 9102 J Clin Oncol 2007 25 1160 1168 10.1200/JCO.2005.04.7118 17401004

[B4] Stahl M Stuschke M Lehmann N Chemoradiation with and without surgery in patients with locally advanced squamous cell carcinoma of the esophagus J Clin Oncol 2005 23 2310 2317 10.1200/JCO.2005.00.034 15800321

[B5] Sobin LH Gospodarowicz MK Wittekind C TNM classification of malignant tumours 2010 7 Chichester, West Sussex, UK; Hoboken, NJ: Wiley-Blackwell

[B6] Lagarde SM ten Kate FJ Reitsma JB Prognostic factors in adenocarcinoma of the esophagus or gastroesophageal junction J Clin Oncol 2006 24 4347 4355 10.1200/JCO.2005.04.9445 16963732

[B7] Lagarde SM ten Kate FJ Richel DJ Molecular prognostic factors in adenocarcinoma of the esophagus and gastroesophageal junction Ann Surg Oncol 2007 14 977 991 10.1245/s10434-006-9262-y 17122988

[B8] Kihara C Seki T Furukawa Y Mutations in zinc-binding domains of p53 as a prognostic marker of esophageal-cancer patients Jpn J Cancer Res 2000 91 190 198 10.1111/j.1349-7006.2000.tb00931.x 10761706 PMC5926332

[B9] Ribeiro U Jr Finkelstein SD Safatle-Ribeiro AV p53 sequence analysis predicts treatment response and outcome of patients with esophageal carcinoma Cancer 1998 83 7 18 10.1002/(SICI)1097-0142(19980701)83:1<7::AID-CNCR2>3.0.CO;2-R 9655287

[B10] Ireland AP Shibata DK Chandrasoma P Clinical significance of p53 mutations in adenocarcinoma of the esophagus and cardia Ann Surg 2000 231 179 187 10.1097/00000658-200002000-00005 10674608 PMC1420984

[B11] Arber N Shapira I Ratan J Activation of c-K-ras mutations in human gastrointestinal tumors Gastroenterology 2000 118 1045 1050 10.1016/S0016-5085(00)70357-X 10833479

[B12] Lievre A Bachet JB Boige V KRAS mutations as an independent prognostic factor in patients with advanced colorectal cancer treated with cetuximab J Clin Oncol 2008 26 374 379 10.1200/JCO.2007.12.5906 18202412

[B13] Schemper M Smith TL A note on quantifying follow-up in studies of failure time Control Clin Trials 1996 17 343 346 10.1016/0197-2456(96)00075-X 8889347

[B14] Bergqvist AS Bergqvist M Brattstrom D Serum p53 autoantibodies as prognostic marker in patients with oesophageal carcinoma Anticancer Res 2001 21 4141 4145 11911308

[B15] Schneider PM Stoeltzing O Roth JA P53 mutational status improves estimation of prognosis in patients with curatively resected adenocarcinoma in Barrett's esophagus Clin Cancer Res 2000 6 3153 3158 10955797

[B16] Shimada H Shiratori T Takeda A Perioperative changes of serum p53 antibody titer is a predictor for survival in patients with esophageal squamous cell carcinoma World J Surg 2009 33 272 277 10.1007/s00268-008-9821-4 19052812

[B17] Takahashi K Miyashita M Nomura T Serum p53 antibody as a predictor of early recurrence in patients with postoperative esophageal squamous cell carcinoma Dis Esophagus 2007 20 117 122 10.1111/j.1442-2050.2007.00656.x 17439594

[B18] Cai HY Wang XH Tian Y Changes of serum p53 antibodies and clinical significance of radiotherapy for esophageal squamous cell carcinoma World J Gastroenterol 2008 14 4082 4086 10.3748/wjg.14.4082 18609695 PMC2725350

[B19] Hainaut P Wiman KG 30 years and a long way into p53 research Lancet Oncol 2009 10 913 919 10.1016/S1470-2045(09)70198-6 19717093

[B20] Hagiwara N Onda M Miyashita M Detection of circulating anti-p53 antibodies in esophageal cancer patients J Nippon Med Sch 2000 67 110 117 10.1272/jnms.67.110 10754600

[B21] Shimada H Takeda A Arima M Serum p53 antibody is a useful tumor marker in superficial esophageal squamous cell carcinoma Cancer 2000 89 1677 1683 10.1002/1097-0142(20001015)89:8<1677::AID-CNCR5>3.0.CO;2-9 11042560

[B22] Angelopoulou K Diamandis EP Sutherland DJ Prevalence of serum antibodies against the p53 tumor suppressor gene protein in various cancers Int J Cancer 1994 58 480 487 10.1002/ijc.2910580404 8056443

[B23] Lubin R Schlichtholz B Teillaud JL p53 antibodies in patients with various types of cancer: assay, identification, and characterization Clin Cancer Res 1995 1 1463 1469 9815945

[B24] Soussi T p53 Antibodies in the sera of patients with various types of cancer: a review Cancer Res 2000 60 1777 1788 10766157

